# Through the Speech and Vocal Signals Hidden Secrets: An Explainable Methodology for Neurological Diseases Early Detection

**DOI:** 10.1007/s41666-025-00213-9

**Published:** 2025-09-02

**Authors:** Patrizia Vizza, Alessio Di Ponio, Giuseppe Timpano, Roberto Bruno Bossio, Giuseppe Tradigo, Giuseppe Pozzi, Pietro Hiram Guzzi, Pierangelo Veltri

**Affiliations:** 1 Department of Surgical and Medical Science, Magna Graecia University, Catanzaro, 88100 Italy; 2 Department of Theoretical and Applied Sciences, University e-Campus, Novedrate, 22060 Italy; 3 Neurological Unit, Azienda Sanitaria Provinciale (AS), Cosenza, 87100 Italy; 4 Department of Electronics, Information and Bioengineering, Politecnico di Milano, Milan, 20133 Italy; 5 Department of Computer Engineering, Modeling, Electronics and Systems (DIMES), University of Calabria, Rende, 87036 Italy

**Keywords:** Neurodegenerative diseases, Neurological biomarkers, Voice analysis, Speech, Machine learning, Explainability

## Abstract

Neurodegenerative diseases progressively damage brain and nervous systems impairing their functionality. Early diagnosis can improve the efficacy of treatments and patient’s life quality. Biomarkers extracted from the human voice can be a simple, efficient, and non-invasive methodology to screen neurodegenerative diseases such as Parkinson’s (PD) and multiple sclerosis (MS). Nevertheless, there is still a lack of reliable and clinically approved methodologies required in large-scale patient applications. We define a methodology for features extracted from voice signals as non-invasive indices for early diagnosis of neurodegenerative diseases. We combine and analyze vowels and speech using a set of machine learning (ML) algorithms trained on a combined set of signal features such as acoustic, articulation, and cepstral ones. The methodology has been fully implemented and applied to a dataset of normophonic and pathological voice signals. Experimental results proved that methodology is able to distinguish healthy from pathological voices, with reliable performances, such as accuracy of 97.5%, sensitivity of 98.5%, precision of 97.0%, F1-score of 98.0%, the Matthews correlation coefficient of 0.95, and AUC of 0.98. Finally, the proposed methodology provides explainability tasks for neurological biomarkers identification from speech and vocal features, confirming its reliability. A github repository with data sample and code is available at https://github.com/PatriziaVizza/SpeechAndVocalSignalsAnalysis.

## Introduction

Parkinson’s disease (PD) and multiple sclerosis (MS) are chronic neurodegenerative disorders characterized by progressive deterioration of neurological function [1]. PD represents the second most common neurodegenerative disease affecting $0.5-1\%$ of people aged between 65 and 69 and increases to $1-3\%$ for people aged over 80. The incidence of multiple sclerosis varies widely geographically, but it is generally estimated to be in the range of 2 $\div$ 15 new cases per 100,000 people per year. Early and accurate diagnosis is needed in both cases (PD and MS) to guarantee successful progression management. Conventional diagnostic methods rely primarily on clinical assessments and neuroimaging, but these approaches often prove unsatisfactory in terms of sensitivity, specificity, and accessibility.

To overcome these limitations, recent contributions explore alternative biomarkers for the early detection of neurodegenerative diseases [2, 3, 4]. The human voice, a complex product of neural activity, offers a promising direction for non-invasive disease assessment [5]. Alterations in vocal characteristics, such as pitch, intensity, and vocal quality changes, may be early indicators of neurodegenerative pathology [6]. Specifically, dysarthria represents a common manifestation of neurological disorders characterized by subtle changes in vocal parameters [7]. In this context, the analysis and classification of human speech combined with single vowel analysis can be considered a non-invasive way of monitoring the progression of the disease [8, 9].

To analyze and classify vocal signals, machine learning (ML) algorithms have been widely used [10], to identify the relationships between changes in vocal characteristics and neurological impairments [11, 12]. ML algorithms learn complex patterns and features associated with different vocal conditions, offering an accurate objective assessment of vocal health that physicians can use in the early detection of vocal disorders. We also propose the use of ML known algorithms as a novel methodology for classifying healthy and neurologically impaired voices, but we focus in particular on two diseases, Parkinson’s (PD) and multiple sclerosis (MS). Moreover, the innovation in the proposed methodology consists of a combined use of vowel pronunciation and speech segments to extract a wide range of biomarkers for PD and ML pathologies. The proposed methodology leverages the ML models that significantly outperform existing approaches in terms of classification accuracy and feature selection. These algorithms have been implemented in an optimized set of Python scripts by means of an innovative workflow protocol. Finally, *explainability ML* techniques have been implemented to validate and reinforce the obtained results in terms of early PD and MS detection, providing valuable insights into mechanisms reflected in the vocal biomarkers.

**Fig. 1:**
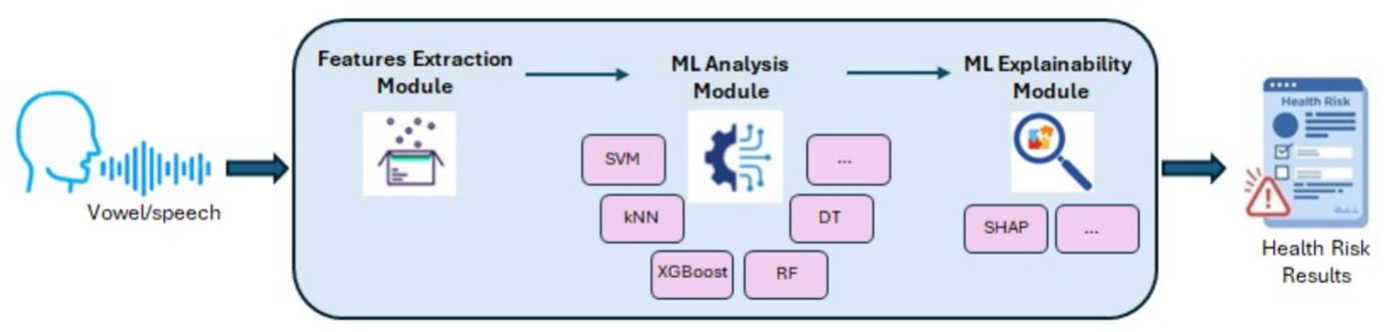
Pipeline of the proposed methodology to report neurological risk indexes. The implemented system processes both speech and vowel signals by extracting relevant features, performs ML analysis and ML explainability, and finally returns a health risk score index. Results explain the correlation between features and (potential) neurological pathology markers

Trained on a dataset of 122 instances, the implemented methodology can learn from additional tests that can also be used to correct indications. After training, the system can be used to screen an incoming patient at potential neurological risk (e.g., family history), as shown in Fig. 1. The system acquires speech and vocal signals and generates both a quantitative risk index and automated alerts for physicians. Additionally, the system provides explainability indications about identified parameters and their link with the presence of suspect indices associated with neurological pathologies. As an additional novelty, an explainability ML pipeline has been setup using the SHAP technique to provide physicians with an interpretation to support the identification of potential biomarkers obtained by the ML-based proposed system. Both ML models and explainability modules have been included in the implemented system (see Fig. 1).

The paper is structured as follows. Section 2 reports the background and state-of-the-art; Sect. 3 details the proposed methodology; Sect. 4 evaluates the results; Sect. 5 discusses the implications of our findings; and finally, Sect. 6 summarizes the key conclusions.

## Background and Related Work

In recent years, voice signal analysis has played an important role in the detection and description of neurodegenerative disorders, through the quantification of relevant signal properties [13, 14, 15, 16]. Voice-based analysis can be performed by phonation and articulation characteristics exploiting different features. The phonatory analysis which involves sustained vowel pronunciation is widely used for its simplicity and ease of data collection. Nevertheless, the study of speech sounds recently offers a more contextualized approach for assessing phonatory function, providing a more comprehensive understanding of vocal production as in [17]. We focus on this by using known techniques in a combined framework applied to vocal and speech analysis.

Machine learning (ML) techniques have been used to enhance features in speech and vocal signal analysis [18, 19, 20]. For example, logistic regression (LR) [21, 22], Extreme Gradient Boosting (XGBoost) [23, 24], random forest (RF) [25, 26], support vector machines (SVMs) [27, 28], convolutional neural networks (CNNs) [29, 30], and *k*-nearest neighbors (kNN) [31, 32] have been used to analyze clinical data to support early detection of neurodegenerative disease [20]. In this direction, ML models can be used to detect changes in vocal and speech patterns indicating neurological disorders and aid the physician in timely diagnosis [33, 34, 35].

The correlation of vocal patterns and speech with single and well-defined aspect of neurological disease, i.e., PD, has been studied in the literature, showing the presence of correlation between vocal patterns and neurological states [36, 37, 38, 39, 41].

Moreover, ML approaches have been extensively evaluated for PD classification, especially on feature selection optimization and classifier performance [41, 42, 43, 44], and comparative analyses between traditional feature-based ML methods and advanced methodological frameworks have also demonstrated promising results in distinguishing both early-stage and advanced PD patients from healthy subjects [45, 46, 47].

Unlike extensive studies regarding the vocal signal in PD characterization, speech-based analysis for multiple sclerosis (MS) diagnosis and monitoring has received less attention. Now, however, this is changing. Contributions in the literature have focused on developing machine learning approaches for the classification and monitoring the progression of the disease through acoustic analysis, demonstrating the effectiveness of speech-based biomarker extraction and classification models for MS diagnosis [34, 48].

Machine learning models are often difficult to interpret due to their complex mathematical structures. While some models, such as decision trees, offer inherent transparency, others, such as random forest, SVM, and XGBoost, are not inherently interpretable, hence the term opaque models [49]. This lack of interpretability is a problem in applications where classification results require explainability. Explainability ML techniques provide explicit and interpretable justifications for their actions, making these models more transparent and reliable for decision makers (e.g., physicians) [50]. Explainable ML techniques help us find which features play the most crucial role in prediction. In several machine learning applications, the rationale behind model predictions is essential for understanding the algorithm’s decision-making process and the significance of the prediction.

To the best of our knowledge, very few references consider a contemporary analysis of multiple neurological diseases as in Illner et al. [51] and the extraction of features in multiple domains (e.g., Quan et al. [52]). Moreover, the use of the explainability approach is still limited, primarily focusing on the binary classification problems of Parkinson’s disease [53, 54]. The simultaneous analysis of different types of pathological voices and the extraction of features in more characterizing domains could represent a breakthrough for differentiating and classifying a wider range of neurological disorders.

In this work, we focus on a novel methodology to discriminate and classify healthy and pathological voices using a comprehensive analysis of vocal biomarkers. The proposed methodology implements machine learning models that significantly outperform existing state-of-the-art methods in terms of classification and feature selection, as measured by standard evaluation metrics. Compared to the literature, our methodology extracts relevant features from multiple domains of vocal signals, including sustained vowels and different speech patterns, enabling discrimination between healthy controls and patients affected by different neurological disorders. Moreover, the SHAP-based model has been integrated to support and explain the decision-making process of the proposed prediction system. This analysis reveals the most relevant vocal biomarkers, improving understanding of the pathophysiology and enhancing the clinical interpretability of the results. We contribute by developing a system covering a broader spectrum of neurological-related pathologies, also including an explainable feature extraction and classification system to process voice-related features. We thus show the potential of using voice-based biomarkers as a valuable tool for the early detection and monitoring of neurological diseases, such as PD and MS.

## The Proposed Methodology

**Fig. 2:**
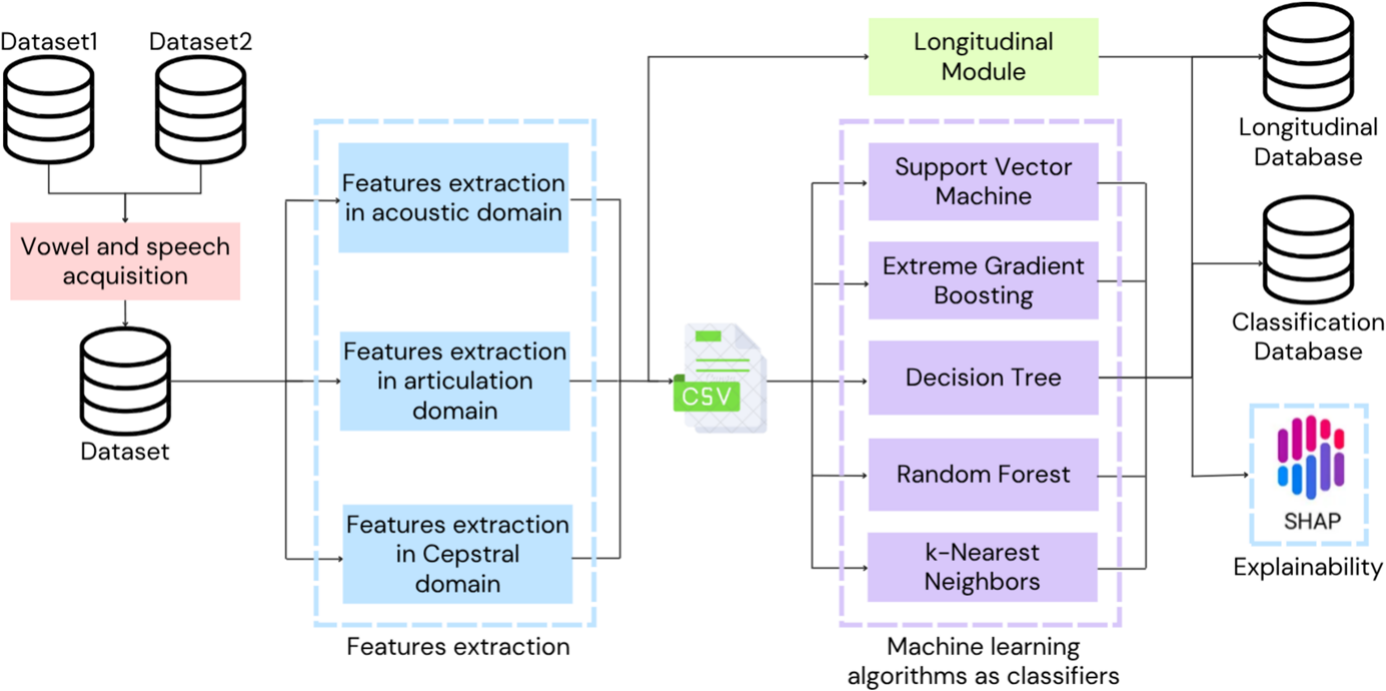
Workflow of the proposed methodology. The major steps are (i) acquisition and pre-processing of vowel and speech signals, (ii) feature extraction from acoustic, articulatory, and cepstral domains, (iii) application of machine learning algorithms for vocal anomaly classification, (iv) utilization of explainable machine learning techniques to interpret model predictions, and finally (v) longitudinal module to study the progression of the disease

The aim of the proposed methodology is to assess neurodegenerative diseases through the analysis of vocal and speech data; this consists of several steps, as depicted in Fig. 2. The first describes acquiring and pre-processing vocal and speech signals from multiple subjects (see Sect. 3.1); the second step extracts the features of the speech from three different domains (i.e., acoustic, articulation, and cepstral domains, in Sect. 3.2); the third deploys different machine learning algorithms for the classification of vocal anomalies (Sect. 3.3); then, the explainability ML approach is used to show how the models predict the final results (Sect. 3.4); and the last step deals with managing, storing, and retrieving longitudinal data for the progression and treatment of the disease in follow-up studies. The longitudinal module integrates capabilities for data acquisition, analysis, storage, classification, and crucially, a follow-up mechanism to enable longitudinal comparisons of changes in extracted vocal features over time by individual patients with Parkinson’s (PD) and multiple sclerosis (MS), monitoring treatment efficacy, and enabling automated patient recall. It acts as an overarching management layer, performing the systematic collection, storage, and analysis of sequential patient data, thereby adding a temporal dimension to the system.

A fully automated Python script of roughly 1500 lines of code is written to perform the entire workflow, from data acquisition and feature extraction to the classification of healthy and pathological voices.

### Dataset Description

We use a database of three different classes of subjects, e.g., healthy subjects (HS), Parkinson’s (PD), and multiple sclerosis (MS), with their related vocal and speech signals (see Table 1). The database includes two different datasets.

The first dataset (Dataset1) is available online [55] and contains voice recordings of patients suffering from Parkinson’s disease. This includes recordings of 65 subjects: 28 subjects are diagnosed with Parkinson’s disease, and the other 37 subjects are healthy. Moreover, this dataset includes different types of vocal signal acquisition: for each subject, sustained vowels, text, sentences, and words have been recorded according to the following protocol [56]:

- Two readings of a phonemically balanced text spaced by a pause (30 s)
- Execution of the syllable /pa/ (5 s), pause (20 s), execution of the syllable /ta/ (5 s)
- Two phonation of the vowel /a/
- Two phonation of the vowel /e/
- Two phonation of the vowel /i/
- Two phonation of the vowel /o/
- Two phonation of the vowel /u/
- Reading of some phonemically balanced words, pause (1 min), and reading of some phonemically balanced phrases

The second dataset (Dataset2) includes healthy subjects and patients affected by multiple sclerosis (MS), both enrolled at the Neurological Operative Unit – Center of Multiple Sclerosis in Cosenza, Italy [57]. Their vocal signals have been stored in an anonymized database. The database contains phonation of vowels /a/, /e/, /i/, /o/, and /u/ recorded for 50 patients affected by MS and for seven healthy subjects (HS), grouped by gender and age. Vocal signal acquisition is performed on a well-defined protocol in agreement with physician specifications, and informed consent signed by each enrolled patient before the acquisition starts. The procedure consists of continuous and sustained vowels (/a/, /e/, /i/, /o/, /u/) phonation for 5 s each one by an omnidirectional microphone (Shure 14A), located approximately 5 cm from the subject lips, with a sampling frequency of 44 kHz, 16-bit resolution, in uncompressed WAV format. The acquisition is made in a laboratory within a controlled acoustic setting and a comfortable environment for the subject.

**Table 1:** The complete database of three different classes of subjects divided by sex and age: healthy subjects (HS), Parkinson’s disease (PD), multiple sclerosis (MS)

Class	Total	Female	Male	Age
HS	44	19	25	19–72
PD	28	9	19	40–80
MS	50	33	19	25–74
Total	122	61	63	19–80

Vocal signals from the two different datasets are consolidated into a unified data Dataset to ensure consistency and efficiency in feature extraction, analysis, and subsequent classification. A custom built Python script systematically extracts patient-specific folders from a designated directory within the collection, generating a structured CSV file. Each recording within the CSV file includes a unique patient identifier (ID) and a compendium of relevant features (see Sect. 3.2).

The complete dataset encompasses 1056 records (720 for vowels and 336 for speech) and 28 features (see Sect. 3.2). Since each feature has a different range of values, data standardization is implemented to enhance comparability. The dataset entails centering the data by subtracting the mean and dividing it by the standard deviation, resulting in a distribution with a mean value equal to zero and a standard deviation equal to one. Moreover, the script deploys a mean imputation strategy to handle missing values; by the scikit-learn library’s SimpleImputer class, the script replaces the null values with the corresponding feature’s mean value.

To address data imbalance, a data augmentation strategy has been adopted. We used time stretching and speed perturbation techniques. To minimize excessive deformation of the original voice signal, stretching/perturbation factors within a narrow range, typically around 0.99 to 1.01, were considered. This approach allowed for an increase in the representation of minority classes while preserving the acoustic integrity of the data.

### Features Extraction

Feature extraction from vocal signals is a critical step in many speech-processing applications. While most traditional methods focus on the frequency-domain (e.g., acoustic features), exploring other domains can help in detecting more complex patterns. For instance, the articulation-domain features provide insights into the shape of the vocal tract, while cepstral-domain features offer a spectral representation that is robust to different types of distortions. By extracting features from these domains, we can achieve a deeper understanding of the acoustic characteristics of the individual’s speech, thereby achieving an improved classification and characterization of speech disorders.

Table 2 depicts the main features extracted from vowel and speech signals in the three different domains, providing a brief description and the number of features considered for each. The total number of extracted features is 28. The identified values are in line with the indications in the literature for neurological diseases.

**Table 2:** Features extracted from vowel and speech signals in the three domains (e.g., acoustic, articulation, and cepstral)

Domain	Main features	Description	Number of features
Acoustic	Fundamental frequency (F0)	Represents the number of times a sound wave produced by the vocal cords is repeated during a given interval of time	1
	Formant frequencies	Frequencies amplified by the vocal tract that provide valuable information about the articulatory movements and the acoustic properties of the speech signal	4
	Jitter	Is mainly influenced by the lack of control of the vibrations of the vocal cords	5
	Shimmer	Changes based on the reduction of glottic resistance and mass lesions on the vocal cords and is correlated with the presence of noisy and breathless emissions	6
	Harmonic-to-noise ratio	Is the measure of the noise of a speech signal and is related to voice quality influenced by irregularities present in the pattern of vibrations of the strings	1
Articulation	Vowel space area (VSA)	Is a two-dimensional representation of the acoustic characteristics of vowels, providing a visual comparison of vowel qualities	2
	Formant centralization ratio (FCR)	Is a normalization factor applied to formant frequencies, crucial for enhancing the sensitivity to vowel centralization and mitigating inter-subject variability	1
Cepstral	Mel-frequency cepstral coefficients (MFCCs)	Detect irregularities or incomplete closure of the vocal folds as well as alterations and turbulences related to vocal fold vibration	2
	Cepstral peak prominence (CPP)	Is a measure of voice quality, particularly robust for the assessment of dysphonia	2
	Zero crossing rate (ZCR)	Represents how many times the signal changes sign divided by the length of the analyzed frame, generally used as an index of the noisiness of a signal	2
	Spectral centroid (SC)	Indicates the weighted mean of the frequencies present in a signal, providing valuable information about the overall brightness or darkness of a sound	2

The main features with related descriptions and the number of extracted features are reported for each domain. The total number of extracted features is 28

#### Features in the Acoustic Domain

Fundamental frequency, formant frequencies, jitter, shimmer, and signal-to-noise ratio (SNR) are extracted as main features in the acoustic domain [58].

The fundamental frequency (F$_0$) is the rate at which the vocal cords vibrate, measured in hertz (Hz). Formant frequencies are prominent peaks in the spectrum of a vocal signal, resulting from resonances within the vocal tract. These frequencies provide valuable information on the articulatory movements and the acoustic properties. Typically, the first four formants (F$_1$, F$_2$, F$_3$, F$_4$) are considered in the vocal signal analysis.

The two parameters jitter and shimmer are calculated to measure any disturbance on the fundamental frequency. The jitter parameter, which is a measure of vocal cord vibration variability, represents the cycle-to-cycle variation in the period of the glottal vibration. The pathological voices are characterized by elevated jitter values. Here, five main jitter metrics are considered:

i. Absolute jitter $J_a$ (1) represents the average difference between consecutive fundamental frequencies.
ii. Relative jitter $J_r$ (2) indicates the ratio of absolute jitter to the average of fundamental frequency.
iii. Relative average perturbation *RAP* (3) consists of the variability of the fundamental frequency with a three-period attenuation factor.
iv. Pitch period perturbation quotient *PPQ*5 (4) represents the variability of the fundamental frequency with a five-period attenuation.
v. Difference of differences of periods *DDP* (5) provides a quantitative measure of period-to-period temporal variations in the voice signal.

In the following equations, $T_i$ and *N* represent the duration expressed in seconds of each period and the number of periods, respectively.

1 $$\begin{aligned} J_a = \frac{1}{N}\sum _{i=1}^{N-1}\left| T_i - T_{i-1} \right| \end{aligned}$$

2 $$\begin{aligned} J_r = \frac{J_a}{\frac{1}{N}\sum _{i=1}^{N-1}T_i} \end{aligned}$$

3 $$\begin{aligned} RAP = \frac{\frac{1}{N-2}\sum _{i=2}^{N-1} \left| \frac{T_{i-1}+T_i+T_{i+1}}{3}-T_i\right| }{\frac{1}{N}\sum _{i=1}^{N-1}T_i} \end{aligned}$$

4 $$\begin{aligned} PPQ5 = \frac{\frac{1}{N-4}\sum _{i=3}^{N-2} \left| \frac{T_{i-2}+T_{i-1}+T_i+T_{i+1}+T_{i+2}}{5}-T_i\right| }{\frac{1}{N}\sum _{i=1}^{N-1}T_i} \end{aligned}$$

5 $$\begin{aligned} DDP = \frac{1}{N-1}\sum _{i=1}^{N-1}\left| T_i - T_{i-1} \right| \end{aligned}$$

The parameter shimmer changes depending on the reduction of glottal resistance and mass lesions on the vocal cords. It represents the cycle-to-cycle variation in the amplitude of the glottal vibration and is correlated with the presence of noisy and breathless emissions. The most commonly used metrics associated with the shimmer considered in this work are as follows:

i. Relative shimmer $S_r$ (6) and its value expressed in dB $S_{db}$ (7) measure the absolute average difference between the amplitudes of two consecutive glottal cycles (vocal periods), divided by the average amplitude.
ii. The *n*-point amplitude perturbation quotient $APQ_n$ shimmer (Eq. 8 with $n = 3, 5, 11$) represents the absolute average difference between the amplitude of a period and the average of the amplitudes of its neighbors (e.g., 3, 5, and 11 neighbors periods), divided by the average amplitude.
iii. Difference of differences of amplitude *DDA* (9) quantifies the long-range correlations in the shimmer signal.

In each equation, $A_i$ refers to the peak-to-peak amplitude of each glottal cycle (vocal period) in the voice signal, and *N* represents the number of periods. Moreover, *k* is the index for neighboring cycles, ranging from $-n/2$ to *n*/2 (excluding 0).

6 $$\begin{aligned} S_r = \frac{\frac{1}{N-1}\sum _{i=1}^{N-1} \left| A_i -A_{i+1} \right| }{\frac{1}{N}\sum _{i=1}^{N-1}A_i} \end{aligned}$$

7 $$\begin{aligned} S_{dB} = \frac{1}{N-1}\sum _{i=1}^{N-1} 20 \log \frac{A_{i+1}}{A_i} \end{aligned}$$

8 $$\begin{aligned} APQ_n = \frac{1}{N} \sum \left| A_i - \frac{1}{n} \sum (A_i - k) \right| \end{aligned}$$

9 $$\begin{aligned} DDA = \frac{1}{N-1}\sum _{i=1}^{N-1}\left| A_i - A_{i-1} \right| \end{aligned}$$

The harmonic-to-noise ratio (HNR), which is the measure of the noise of a speech signal, is related to the voice quality (10). HNR is influenced by irregularities in the pattern of vibrations of the vocal strings, such as the breaking of the voice and the disturbance of the frequency or amplitude.

10 $$\begin{aligned} HNR = 10 \log (\frac{NoiseEnergy}{HarmonicEnergy})dB \end{aligned}$$

To extract the features in the acoustic domain, we use the Parselmouth library in Python to provide a complete interface to the internal code of the open-source software Praat [59], performing the audio analysis of the voice and the speech in the field of phonetics.

#### Features in the Articulation Domain

The vowel space area (VSA) is a two-dimensional representation of the acoustic characteristics of vowels, typically plotted on a graph where the first formant frequency (F$_1$) is on the horizontal and the second formant frequency (F$_2$) is on the vertical axis [60]. This graphical representation provides a visual comparison of vowel qualities and can be used to assess various speech-related phenomena such as the impact of pathological conditions on speech production.

Three primary features are commonly employed to quantify VSA:

i. The triangular vowel space area (tVSA), computed as the area of the triangle formed by the F$_1$ and F$_2$ coordinates of the vowels /i/, /u/, and /a/ (11).
ii. The quadrilateral vowel space area (qVSA) is the area of the quadrilateral defined by the F$_1$ and F$_2$ coordinates of the vowels /i/, /u/, /e/, and /a/ (12). Both tVSA and qVSA are typically computed by the Euclidean distance.
iii. The formant centralization ratio (FCR) is a normalization factor applied to formant frequencies, crucial for enhancing sensitivity to vowel centralization and mitigating inter-subject variability (13).

11 $$\begin{aligned} tVSA = \frac{1}{2}(F_1^i(F_2^a-F_2^u)+F_1^a(F_2^u-F_2^i)+F_1^u(F_2^i-F_2^a)) \end{aligned}$$

12 $$\begin{aligned} {\begin{matrix} qVSA & = \frac{1}{2}((F_1^eF_2^i+F_1^aF_2^e+F_1^uF_2^a+F_1^iF_2^u)-\\ & (F_1^iF_2^e+F_1^eF_2^a+F_1^aF_2^u+F_1^uF_2^i)) \\ \end{matrix}} \end{aligned}$$

13 $$\begin{aligned} FCR = \frac{F_2^u + F_2^a +F_1^i + F_1^u}{F_2^i + F_1^a} \end{aligned}$$

#### Features in the Cepstral Domain

The cepstral^1^ domain features have proven to be invaluable in the analysis due to their ability to provide a robust and informative representation of the underlying acoustic characteristics of speech.

The Mel-frequency cepstral coefficients (MFCCs) are a widely deployed technique for extracting features from the vocal tract in audio signals. The computation of MFCCs involves five primary stages: (i) signal framing, (ii) power spectrum calculation, (iii) application of a Mel-filterbank, (iv) logarithmic transformation, and (v) the discrete cosine transform (DCT). MFCCs are particularly effective in detecting irregularities or incomplete closure of the vocal folds. Alterations related to vocal fold vibration are often evident in the lower MFCC bands, while turbulence components are more pronounced in the higher ones.

The cepstral peak prominence (CPP) is a measure of voice quality, particularly robust for the assessment of dysphonia. CPP quantifies the amplitude of the cepstral peak by comparing the level of harmonic organization in the voice recording to the background noise caused by respiration. Thus, it detects the lack of breathiness in patients, both in sustained vowel production and in phrases.

The zero crossing rate (ZCR) represents how many times the signal changes sign (from positive to negative and vice versa) divided by the length of the analyzed frame. ZCR is often used as an index of the noisiness of a signal. Generally, high values are detected in very noisy signals.

The spectral centroid (SC) is a measure of the center of mass of the spectrum, indicating the weighted mean of the frequencies present in a signal. In the context of voice analysis, SC provides valuable information on the overall brightness or darkness of a sound.

For each cepstral feature, we compute two metrics (e.g., mean and median values) by using the Librosa library in Python through the implementation of the *mfcc*() and *flatten*() functions. To compute the CPP metric, we develop a function named *cppDef* based on the following procedure: (i) a high pass filter applied to the vocal signal, to suppress unwanted low frequencies; (ii) the signal divided into frames, and then Hanning’s window is applied to reduce the effects of discontinuity at the edges of the single frame; (iii) the cepstrum of the vocal signal is computed and the peak of the signal in the allowed frequency range found; (iv) lastly, the final value is normalized.

### Classifiers

Support vector machine (SVM), Extreme Gradient Boosting (XGBoost), decision tree (DT), random forest (RF), and *k*-nearest neighbor (kNN) are applied as machine learning models for the classification of vocal signals.

Every model requires data for training, i.e., defining the parameters inside every model, and some data for validation, i.e., measuring the performances of the previously trained model.

We apply the *k*-fold stratified cross-validation strategy to evaluate and compare the applied models [61, 62]. This technique consists of randomly dividing the training dataset into *k* smaller groupings without reinsertion. $k-1$ groupings are used to train the model and to evaluate its performance. This procedure is repeated *k* times to obtain *k* models as well as *k* performance estimates. With respect to the standard approach, this method searches for the same proportions between the classes in each grouping thereby reducing bias and variance. In this work, a ten-fold stratified cross-validation is applied.

The $scikit-learn$ library in Python has been used to implement the clustering, the regression, and the classification algorithms. To apply machine learning models, the dataset has been divided into 30% for data testing (e.g., 317 records) and 70% for data training (e.g., 739 records).

#### Support Vector Machine

Support vector machine (SVM) is a supervised learning algorithm designed to find the hyperplane that best separates the data points of one class from those of another. This model is optimized to mitigate overfitting by reducing the dimensionality of the features through the tuning of the hyperparameters (e.g., type of kernel function, parameters tuning, gamma, polynomial degree, class weight, and probability). Hyperparameter optimization is implemented by the grid search optimization technique [63]. This technique executes an exhaustive search, evaluating all the possible combinations of the hyperparameter values within a specified range. The grid search suggests using a moderate penalty for classification errors, no class weighting, a very low gamma value (0.1), and a radial basis function (RBF) kernel. Once optimized, the decision regions are estimated. The dataset is compressed through principal component analysis (PCA), minimizing information loss while reducing dimensionality.

#### Extreme Gradient Boosting

Extreme Gradient Boosting (XGBoost) is a powerful machine learning algorithm that employs a gradient boosting framework to iteratively construct an ensemble of decision trees and strong predictive models. XGBoost iteratively builds an ensemble of weak base learners, typically decision trees, by minimizing a differentiable loss function. In each iteration, the algorithm focuses on predicting the residuals of the previous ensemble, effectively correcting shortcomings and iteratively improving its ability to fit the training data. The integration of this approach with optimized computational methods, as well as its efficiency and scalability, contributes to making XGBoost a highly regarded algorithm in both classification and regression tasks, characterized by rapid computation, predictive accuracy, and robust generalization. In this case, to implement XGBoost, binary entropy is chosen as the evaluation metric due to its sensitivity to the model’s ability to discriminate between classes. To prevent overfitting and improve generalization, a maximum depth of 6 is set for the decision trees. Then, a learning rate of 0.1 is chosen to allow for gradual updates of the model’s parameters and improve generalization to unseen data.

#### Decision Trees

Decision trees (DT) are predictive models used to classify data. The tree is constructed by recursively partitioning the data based on the feature that maximizes the information gain. Some hyperparameters like maximum depth, maximum number of leaves, and minimum sample split are tuned to prevent overfitting and improve generalization. The maximum depth controls the overall size of the tree: greater depths can capture more complex relationships in the training data, but increase the risk of overfitting. The maximum number of leaves specifies the maximum number of terminal nodes in the tree: limiting the number of leaves simplifies the model and reduces overfitting. The minimum sample split determines the minimum number of samples required to split an internal node: increasing this value can make the model more robust to noise, but an excessively high value might prevent capture of all the relationships in the data. Here, we apply 8 depth levels, a maximum number of 23 leaves, and a minimum of 2 samples. Finally, entropy is used as the splitting criterion to minimize impurity in the resulting partitions.

#### Random Forests

Random forest (RF) is an ensemble method that gained significant prominence in machine learning due to its robustness to overfitting. The model constructs multiple decision trees and aggregates their predictions, effectively reducing variance and improving generalization. Random forests are less intuitive to interpret than single decision trees, but less sensitive to noise, simplifying the tuning of the hyperparameter. Using grid based search optimization techniques we optimize the following hyperparameters:

(i) Number of trees, i.e. large number of trees improves performances but it increases computational cost.
(ii) Bootstrap sample size, i.e., sample size can imply diversity among trees, still influencing overall performances.
(iii) Features number, i.e., a smaller subset of features can improve generalization.

For this application, four random features for each split and an optimal value of 40 trees are determined by the grid search technique. Moreover, the same sample size as the original dataset is used.

#### *k*-Nearest Neighbors

*k*-nearest neighbor (kNN) is a non-parametric, lazy learning algorithm, which stores the entire training dataset and classifies new data points based on the majority class of its *k*-nearest neighbors. This approach offers the advantage of adaptability to new training data, but can be computationally expensive for large datasets. We set the following key hyperparameters: (i) number of neighbors (*k*) set to 10, determining the size of the neighborhood for classification; (ii) metric chosen as the Manhattan distance to calculate the distance along orthogonal axes [64]; (iii) influence of neighbors based on distance (e.g., closer points having a greater impact due to their inversely proportional weight assignment); (iv) leaf size set to 20, defining the minimum number of data points in a leaf node.

### Explainable ML Models

Explainability techniques address human limitations in grasping the complexities of sophisticated models [65]. Explainability algorithms are generally divided as ante hoc and post hoc: ante hoc methods prioritize interpretability during model design; post hoc methods analyze trained models using techniques to explain their behavior [66, 67]. Complex models, including deep neural networks and sophisticated machine learning algorithms, often require post hoc techniques to explain their behavior. These techniques can be used to understand the model’s overall functioning or explain individual predictions [68].

Given the different types of machine learning models deployed, from intrinsically interpretable ones like decision trees (DT) to more complex models like gradient boosting (GB) and support vector machine (SVM), we chose here model-agnostic post hoc explainability techniques. This approach ensures uniform explainability across all the models tested on the extracted data.

The literature reports several model-agnostic techniques for model explainability, including Local Interpretable Model-agnostic Explanations (LIME), SHapley Additive exPlanations (SHAP), Partial Dependence Plots (PDP), and Individual Conditional Expectation (ICE) plots: these techniques represent the effects of input variables on model predictions for both global and local interpretation [69]. We choose here the SHAP technique to explain the ML models due to its ability to provide global explainability by analyzing the average feature contributions to the model’s decisions. Moreover, SHAP can also perform local explainability, examining the contribution of each feature to one single instance of the dataset.

SHAP is based on the calculation of Shapley values, which assess the contribution of each feature by computing the marginal impact of each feature exerted on the model predictions. This evaluation considers all the possible combinations of features [50, 68]. The SHAP technique involves training the model, denoted as $f_{S \cup \{ i \}}$, using the features in subset *S* and one additional feature *i*. Afterwards, a further model, denoted as $f_{S}$, is trained only on the features in *S*. The comparison between the two models is computed through the difference in their output when an observation $x_S$ is provided as input (i.e., the values of the features in subset *S*). This operation is repeated for all the possible subsets *S* within the set of features *F*, excluding *i* ($S \subseteq F \setminus \{ i \}$), thus calculating the Shapley value $\phi _i$ for feature *i* (14) as a weighted average of all these calculated differences [68]:

14 $$\begin{aligned} \phi _i = \sum _{S \subseteq F \setminus \{i\}} \frac{|S|! \, (|F| - |S| - 1)!}{|F|!} \, \big [ f_{S \cup \{i\}}(x_{S \cup \left\{ i \right\} }) - f_S(x_S) \big ] \end{aligned}$$

However, calculating SHAP values for each feature can be computationally expensive. For this reason, the classic implementation of SHAP involves the use of kernels that approximate the Shapley values. Several types of kernels have been developed, each for specific model categories (e.g., SHAP kernels, TreeSHAP, DeepSHAP, LinearSHAP) [68, 70].

Using Python’s SHAP library [71], we quantify the impact of each feature on the model’s predictions. For every individual observation, we analyze the global summary plot, showing overall feature contributions as well as the local force plots; the aim of this is to understand the impact of features for healthy and unhealthy subjects.

## Results

Here, the results of deploying the classification models (i.e., described in Sect. 3.3) to the computed parameters are analyzed (i.e., described in Sect. 3.2) over the considered data (i.e., described in Sect. 3.1). Then, the explainability results for both local and global explanations achieved by deploying the SHAP model are presented (i.e., described in Sect. 3.4).

### Performance Metrics

When evaluating the efficacy of a method in detecting a pathology, four possible outcomes are expected: (i) a true positive (TP), the ill subject is correctly identified as ill; (ii) a false negative (FN), the ill subject is misclassified as healthy; (iii) a true negative (TN), the healthy subject is correctly classified as healthy; and (iv) a false positive (FP), the healthy subject is misclassified as ill.

The following evaluation metrics are used to evaluate the performance of classification models in machine learning: (i) accuracy, (ii) sensitivity, (iii) specificity, (iv) precision, (v) F1-score, and (vi) Matthews correlation coefficient (MCC). These metrics provide different perspectives on how well a model can distinguish between different classes. These metrics are scaled to a dimensionless range of [0,1], where an outcome of 0 denotes the lowest achievable performance and 1 represents the highest. MCC ranges from [$-$1,1], where $-$1 indicates total disagreement and +1 signifies perfect prediction.

Accuracy (Acc) is the proportion of correct predictions (both TP and TN) from the total number of cases (15).

15 $$\begin{aligned} Acc=\frac{TP+TN}{TP+TN+FP+FN} \end{aligned}$$

Sensitivity (Sens), also known as true positive rate or recall, measures the proportion of actual positives that are correctly identified (16).

16 $$\begin{aligned} Sens=\frac{TP}{TP+FN} \end{aligned}$$

Specificity (Spec), also known as true negative rate, measures the proportion of actual negatives identified correctly (17).

17 $$\begin{aligned} Spec=\frac{TN}{TN+FP} \end{aligned}$$

Precision (Prec) is also known as positive predictive value and measures the proportion of correct positive predictions (18).

18 $$\begin{aligned} Prec=\frac{TP}{TP+FP} \end{aligned}$$

The F1-score (F1) is the harmonic mean of precision and recall, providing a single metric that balances both (19).

19 $$\begin{aligned} F1=2*\frac{Prec*Sens}{Prec+Sens} \end{aligned}$$

The Matthews correlation coefficient (MCC) measures the quality of predictions representing the correlation between the predicted and actual class labels (20). It ranges from $-$1 to +1, indicating total disagreement or perfect prediction, respectively.

20 $$\begin{aligned} MCC=\frac{TP*TN-FP*FN}{\sqrt{(TP+FP)(TP+FN)(TN+FP)(TN+FN)}} \end{aligned}$$

Lastly, the confusion matrix is a table providing a detailed summary of the performance of the predictive model, showing the counts of TP, TN, FP, and FN. The receiver operating characteristic (ROC) curve is a graphical plot depicting the diagnostic ability of a binary classifier system as its discrimination threshold is varied. This curve is generated by plotting the true positive (TP) against the false positive (FP) at different threshold settings. The area under the ROC curve (AUC) is a single value that summarizes the performance of the model (i.e., the higher AUC, the better the model).

### Classification Results

The proposed methodology has been tested by the stratified *k*-fold cross-validation technique and assessing the performance metrics described in Sect. 4.1.

Table 3 presents the classification performance of the five machine learning algorithms considered: support vector machine (SVM), Extreme Gradient Boosting (XGBoost), decision tree (DT), random forest (RF), and *k*-nearest neighbors (kNN). The best-performing model for each metric is highlighted in bold. According to Table 3, the best performance metric values in bold for vocal-based PD and MS detection are accuracy = 97.5%, specificity = 99.0%, precision = 97.0%, F1-score = 0.98, and MCC = 0.95 obtained with SVM as well as sensitivity = 98.5% and ROC = 99% achieved by SVM.

Table 4 reports the confusion matrices of the five different classification models. Each matrix compares the labels predicted by the model against the true labels of the data. SVM and XGBoost perform similarly with the highest number of classes correctly classified and with slightly fewer false positives than DT, RF, and kNN. kNN exhibits the highest rate of false positives while DT and RF show slightly higher false negatives than the other.

**Table 3:** Classification performance metrics of the five machine learning algorithms (i.e., SVM, XGBoost, DT, RF, and kNN) with the optimal metric values highlighted in bold

ML algorithm	Performance metrics						
	Accuracy	Sensitivity	Specificity	Precision	F1-score	MCC	ROC
SVM	0.97	**0**.**99**	0.98	0.97	0.98	0.93	**0**.**99**
XGBoost	**0**.**98**	0.95	**0**.**99**	**0**.**97**	**0**.**98**	**0**.**95**	0.97
DT	0.94	0.95	0.94	0.95	0.95	0.87	0.92
RF	0.96	0.98	0.98	0.95	0.98	0.90	0.98
kNN	0.93	0.97	0.92	0.97	0.95	0.87	0.98

SVM and XGBoost achieve the highest performance for vocal-based disease detection. SVM shows a sensitivity of 0.99 and an ROC of 0.99. XGBoost presents an accuracy of 0.98, specificity of 0.99, precision of 0.97, F1-score of 0.98, and Matthews correlation coefficient (MCC) of 0.95

**Table 4:** Confusion matrices for each of the five classification models are reported, showing the comparison between predicted and true labels

(a) Confusion matrix for SVM				
		Predicted label		
		0	1	Total
True label	0	113	7	120
	1	3	194	197
	Total	116	201	317
(b) Confusion matrix for XGBoost				
		Predicted label		
		0	1	Total
True label	0	114	6	120
	1	2	195	197
	Total	116	201	317
(c) Confusion matrix for DT				
		Predicted label		
		0	1	Total
True label	0	111	9	120
	1	10	187	197
	Total	116	201	317
(d) Confusion matrix for RF				
		Predicted label		
		0	1	Total
True label	0	109	11	120
	1	4	193	197
	Total	116	201	317
(e) Confusion matrix for kNN				
		Predicted label		
		0	1	Total
True label	0	115	5	120
	1	15	182	197
	Total	116	201	317

SVM and XGBoost demonstrate comparable results with the most accurate class predictions and marginally fewer false positives compared to DT, RF, and kNN, whereas kNN has the highest false positive rate, and DT and RF exhibit a slightly increased number of false negatives

**Fig. 3:**
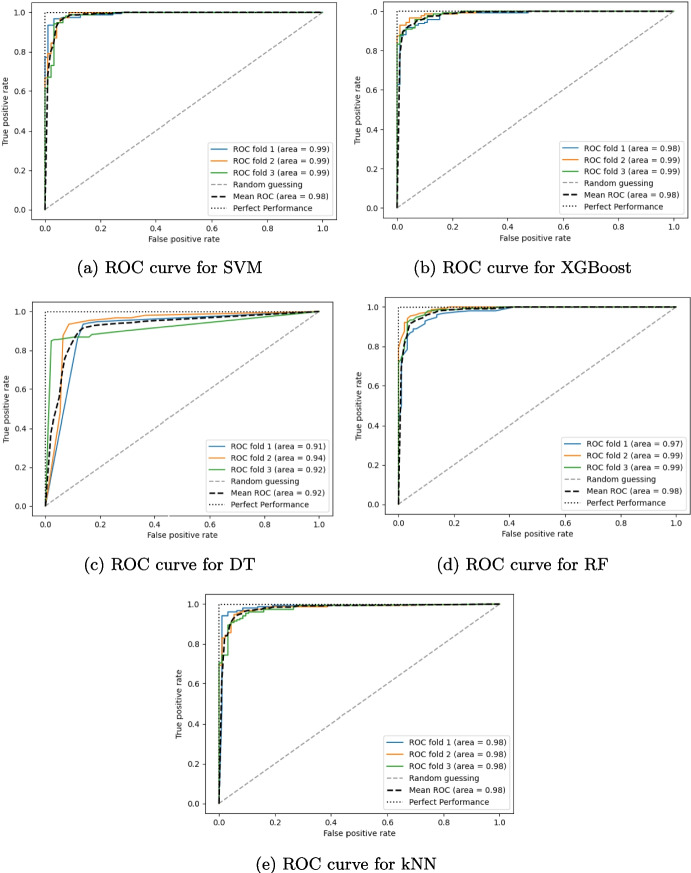
Receiver operating characteristic (ROC) curves are displayed to visually compare the performance of the five classifiers, i.e., SVM (**a**), XGBoost (**b**), DT (**c**), RF (**d**), and kNN (**e**). The area scores are also calculated via stratified three-fold cross-validation to quantify their performance. All models exhibit high area values with SVM and XGBoost demonstrating superior discrimination, while DT, though performing well, presents a slightly lower score, suggesting a potential for overfitting or the need for further hyperparameter optimization

Figure 3 shows the receiver operating characteristic (ROC) curves for each classifier, so as to provide a visual comparison of their performances. The area under the curve (AUC) of the ROC curve represents a quantitative measure of the performance of the classifier. In this study, the AUC is calculated by a stratified three-fold cross-validation. The area scores for all the models are consistently high, indicating excellent overall performance. SVM shows the highest average AUC, closely followed by XGBoost, RF, and kNN. The curves of SVM and XGBoost are closest to the ideal point (0,1), implying a marked ability to discriminate between positive and negative classes. DT has a slightly lower but still acceptable AUC; this indicates that it is slightly more prone to overfitting or might benefit from additional hyperparameter tuning. Based on the ROC curves, all four models perform well as regards classification. However, SVM and XGBoost appear to have a slight edge in terms of overall accuracy and robustness.

Results prove that SVM and XGBoost can achieve higher specificity and sensitivity values, along with competitive precision and F1-score. Their discriminative power is further evidenced by robust ROC AUC values, indicating a remarkable capability to distinguish between positive (e.g., PD and MS) and negative (e.g., healthy) classes. Overall, DT and kNN yielded the poorest performance of the evaluated classifiers, even if the kNN algorithm exhibits the highest prevision value. Concerning DT, deploying an ensemble approach with random forest significantly improves performance metrics, making RF a viable alternative to the SVM and XGBoost.

#### Multi-class Classification

**Fig. 4:**
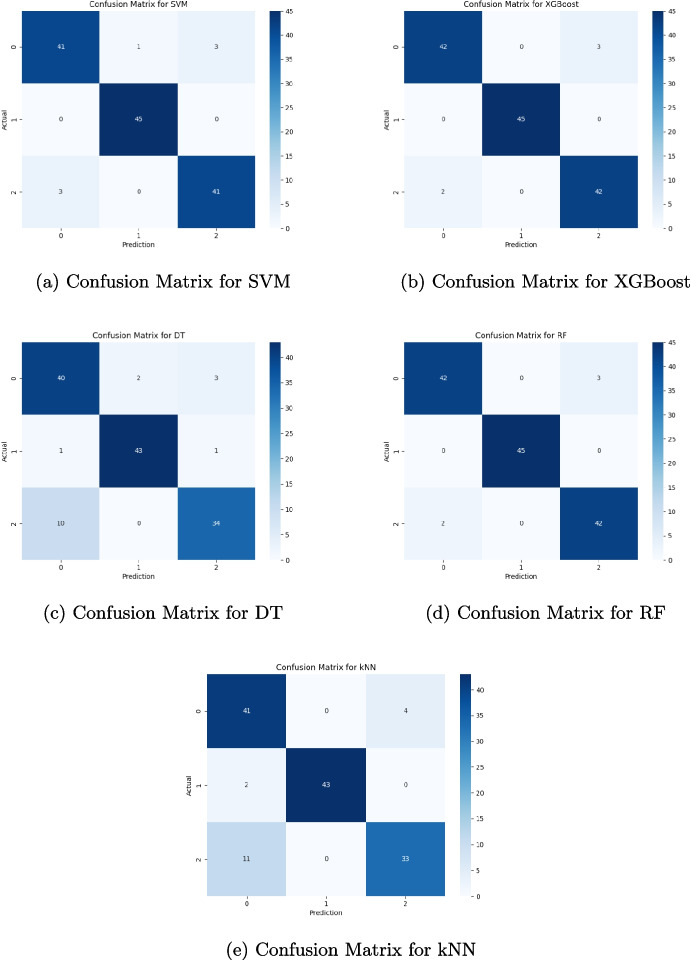
Confusion matrices for each of the five classification models are reported, showing the comparison between predicted and actual labels. RF and XGBoost demonstrate comparable performance with the most accurate class predictions, where all records of class 1 (i.e., Parkinson) have been associated to the correct class. DT and kNN have the highest false rate. SVM presents a slightly different behavior to RF and XGBoost

The multi-class classification was performed on a balanced dataset of 670 vocal records acquired by 45 healthy subjects (class 0), 45 Parkinson patients (class 1), and 44 multiple sclerosis subjects (class 2). To apply machine learning models, the dataset was divided in two groups: 20% of the vocal signals have been used for data testing (e.g., 134 records) and 80% for data training (e.g., 536 records). Table 5 presents the classification performance metrics of the five machine learning algorithms considered: support vector machine (SVM), Extreme Gradient Boosting (XGBoost), decision tree (DT), random forest (RF), and *k*-nearest neighbors (kNN). Figure 4 reports the confusion matrices of the five different classification models. Both performance metrics and confusion matrix results demonstrate the ability of the proposed methodology to perform a multi-class classification, with a high capability to annotate the different pathological voices (e.g., Parkinson and multiple sclerosis) w.r.t. the healthy voices. We conclude claiming that even using the multi-class methodology, different pathological conditions are still distinguished with performances that are above 0.9 values. This demonstrates that the methodology proposed herein is robust and efficient.

**Table 5:** Classification performance metrics of the five machine learning algorithms (i.e., SVM, XGBoost, DT, RF, and kNN) for multi-class classification

ML algorithm	Performance metrics						
	Accuracy	Sensitivity	Specificity	Precision	F1-score	MCC	ROC
SVM	0.95	0.95	0.97	0.95	0.95	0.92	0.99
XGBoost	0.96	0.96	0.98	0.96	0.96	0.94	1.00
DT	0.87	0.88	0.94	0.88	0.87	0.81	0.95
RF	0.96	0.96	0.98	0.96	0.96	0.94	0.99
kNN	0.87	0.87	0.94	0.88	0.87	0.81	0.98

RF and XGBoost achieve the highest performance for vocal-based disease detection. RF shows a sensitivity of 0.99 and an ROC of 0.99. XGBoost presents a specificity of 0.98 and an ROC value of 1.00

#### Deep Learning Approach for Multi-class Classification

**Fig. 5:**
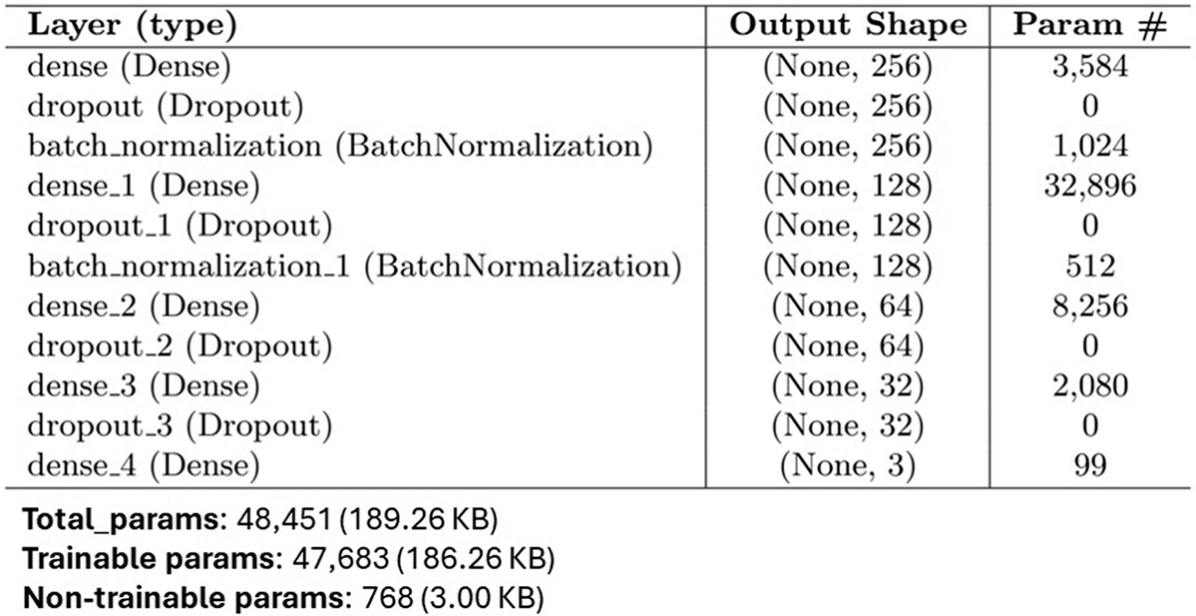
Deep-learning architecture of the proposed model. The architecture is composed of a series of layers which, starting from the input one on top (i.e. “dense”) extract information from input audio files and, in the output layer at the bottom (i.e. “dense_4”, having 3 neurons), predict the probability of their 3 class (i.e., 0 healthy, 1 parkinson, 2 multiple sclerosis)

**Fig. 6:**
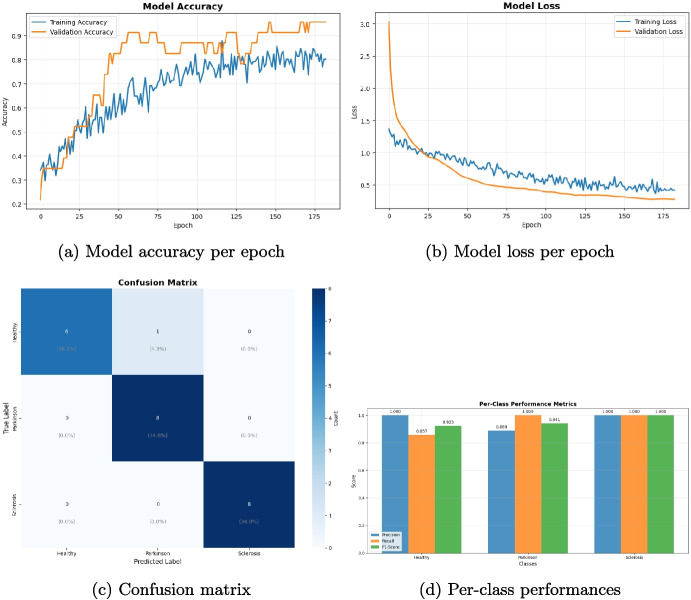
Deep model performances in terms of accuracy and loss for both training and test, confusion matrix related to the test set, and overall per-class performance of the model

We enriched the testing evaluation of the proposed methodology by exploring a deep learning architecture based on a deep neural network, whose structure is depicted in Fig. 5. The total parameters of the model are more than 48k, 47,683 of which are trainable. The output layer (dense_4 in Fig. 5) is a dense layer having in input 99 weights (Param # in Fig. 5) and three output neurons, each activated in the presence of an input audio file of the corresponding class. As with the previously described experiments, here, we also used balanced classes with a total of 114 records, which have been split with a 20% threshold into two training and test sets. Figure 6 shows deep model performances in terms of accuracy and loss for both training and validation test, the confusion matrix related to the test set, and the overall per-class performance of the model. For the training phase, we chose to set 200 epochs, but the process stopped at Epoch 183 for an early stopping condition. The best weights obtained at epoch 168 were chosen. The total training accuracy of the model is 0.8571, while the test accuracy is 0.9565. The per-class performances of the deep network are as follows:

- (i) Healthy: precision = 1.000, recall = 0.857, f1 = 0.923, support = 7
- (ii) Parkinson: precision = 0.889, recall = 1.000, f1 = 0.941, support = 8
- (iii) Sclerosis: precision = 1.000, recall = 1.000, f1 = 1.000, support = 8

Results have been compared with similar deep learning applications for early detection of Parkinson’s and the reported performance indexes are comparable with the here presented ones [72].

### Explainability Results

The explainability approach is used to understand the key features influencing individual patients’ prediction of health/disease. The SHAP algorithm implemented in Python generates graphs for feature evaluation. These graphs enhance the model’s understanding and reliability, enabling the clinician to make a more informed diagnosis.

We then analyze the graphs related to the local and global explainability to evaluate the impact of the features locally on the individual predictions and globally on the final classification of the model, respectively. Next, the local and global results regarding the XGBoost model, which achieves the highest performance metrics, are reported.

#### Local Explanations

**Fig. 7:**
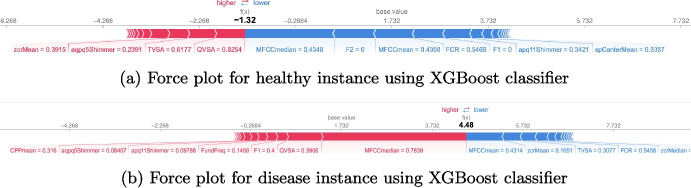
Example of force plots for local explainability in healthy (**a**) and disease (**b**) instances. In **a**, a final prediction of $-$1.32, below the base value of 1.732, is shown, resulting in a healthy classification. In **b**, a prediction of 4.48, significantly above the base value, indicates a disease classification. Both force plots highlight MFCCmedian, MFCCmean, and QVSA as key features, demonstrating their influential positive and negative contributions to the classification

Using force plots from the SHAP library, we compute local explainability: Fig. 7 provides an example. The force plots help us to understand the model’s decisions for individual predictions, by providing useful information on the features that are most significant in the decision-making process, an essential step in validating the model and obtaining clinical insights from the data.

Figure 7a and b highlight the local explainability of classifications for healthy and unhealthy subjects, respectively. The graphs depict the features that drive the instance’s classification towards a particular class (e.g., health or disease), w.r.t. to the baseline value. This value represents the average prediction of the model on the training dataset: the goal of local explainability is to understand how each feature modifies this baseline leading to the final prediction for the specific instance.

In Fig. 7a, the final prediction value $f(x)=-1.32$ is lower than the base value of 1.732 indicating that the instance will be classified in the healthy class. In Fig. 7b, instead, the final prediction value $f(x)=4.48$ is significantly higher than the baseline value, indicating that the instance will be classified in the disease class. The two graphs indicate that features such as MFCCmedian, MFCCmean and QVSA play a prominent role in the classification process, introducing both positive and negative contributions, which suggests that the intrinsic values of these features seem to be determinants for classifying both healthy and unhealthy subjects.

**Fig. 8:**
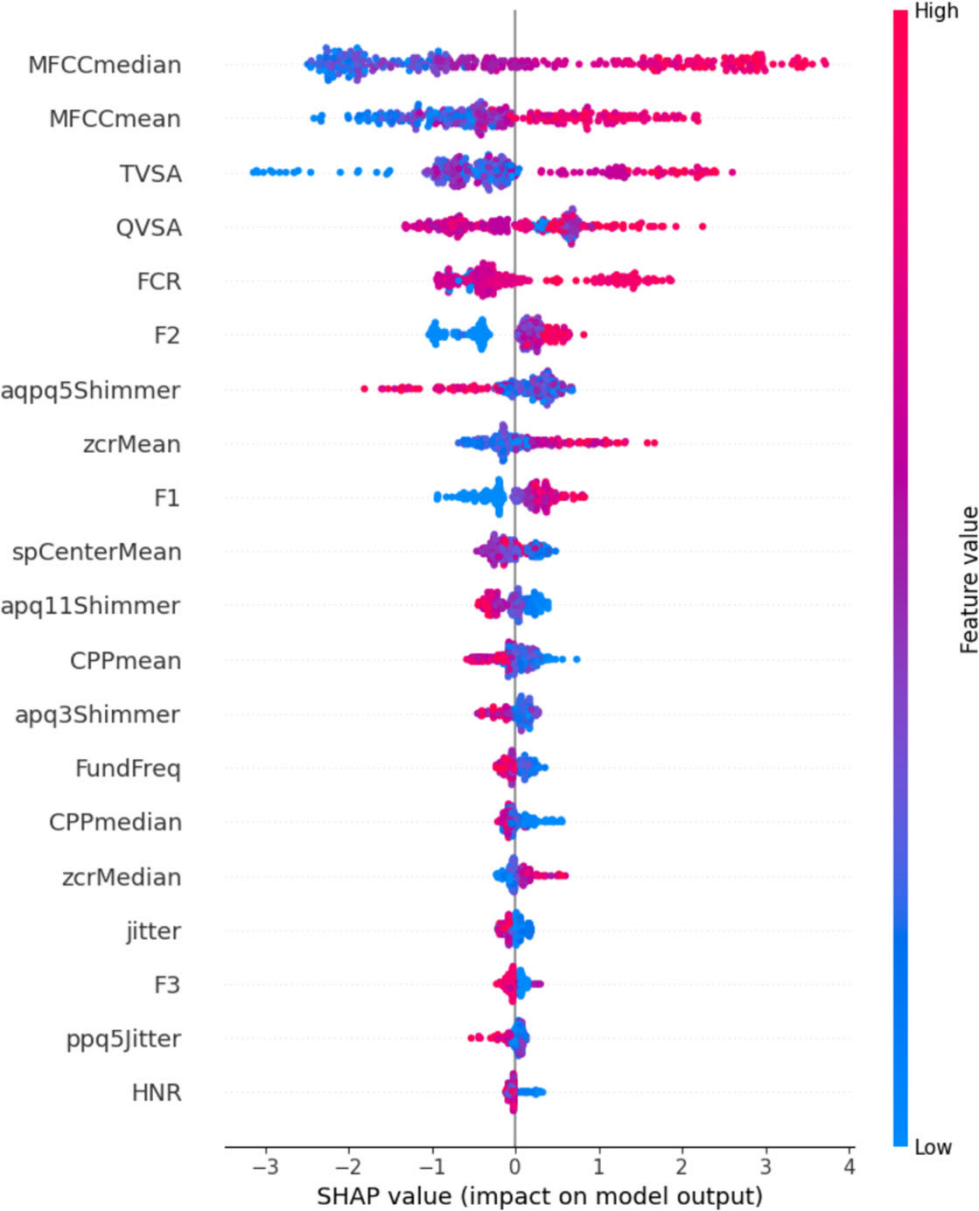
SHAP summary plot for global explainability. MFCC median and MFCCmean values strongly indicate neurological pathologies, while TVSA and QVSA, though less influential, require local analysis due to their variable SHAP values, with blue (positive) SHAP values signifying pathological predictions and red (negative) values indicating healthy predictions

#### Global Explanations

Global explainability enables the identification of the features that most significantly impact the classification model’s decisions, which allows to identify the most important features from the vocal signals of both neurodegenerative diseases (MS and PD) that have the greatest computational and biological relevance on the final classification.

Figure 8 shows a SHAP summary plot, which provides a global view of the features that most influence the predictions. On the vertical axis, the features are ordered according to their influence on the final prediction; on the horizontal axis, the SHAP values are reported, indicating the impact of each feature on the model’s prediction. Positive SHAP values in blue and negative SHAP values in red move the prediction toward the pathological and healthy classes, respectively. Features such as MFCCmedian and MFCCmean are crucial for distinguishing between healthy and pathological subjects: increased values of these features strongly suggest the presence of a pathological condition. Furthermore, features such as TVSA and QVSA also significantly influence predictions, albeit to a lesser extent than the MFCC coefficients. The variability of the SHAP values of these features highlights that their behavior differs between individual observations, so a local analysis and validation are important to correctly interpret the specific predictions.

## Discussion

In recent years, machine learning techniques have gained ever greater attention in diagnosing neurological disorders, such as Parkinson’s disease (PD) and multiple sclerosis (MS), by exploring and analyzing vocal biomarkers to distinguish between healthy and pathological individuals. Deployed machine learning techniques include support vector machines, random forests, and neural networks. The main aim of most studies is to classify PD based on the acoustic features extracted from vocal recordings, e.g., jitter, shimmer, and harmonic-to-noise ratio (HNR).

A comparative analysis of the literature reveals both common trends and disparities in the application of machine learning to vocal-based Parkinson’s disease (PD) and multiple sclerosis (MS) classification. Features extracted in the cepstral domain, e.g., Mel-frequency cepstral coefficients (MFCCs), emerge consistently as a powerful feature set in characterizing vocal changes associated with PD. Nonetheless, the best feature selection may vary w.r.t specific stage or severity of the disease and the chosen machine learning technique. Moreover, studies in the literature report different levels of classification accuracy, with some techniques achieving commendable sensitivity and specificity, while others encounter difficulties in differentiating subtle vocal variations.

Direct comparisons between the methodology proposed here and the existing approaches are problematic due to the variations in the datasets, the methodologies, and the population of subjects. Nonetheless, Table 6 depicts a comparative analysis of ML algorithms, extracted features, and best performance metrics reported in the recent literature, concerning PD and MS detection studies w.r.t. the proposed methodology. References for the comparison are reported in Sect. 2.

**Table 6:** A comparative analysis of the proposed methodology versus recent literature, focusing on the machine learning algorithms, extracted features, and performance metrics employed in PD and MS detection

Author, year	Dataset	Machine learning algorithms	Number of features	Accuracy	Specificity	F1-score
Svodoba et al. [34]	66 HS	RF, kNN, SVM, XGBoost	12	0.82	0.75	-
	65 MS	Gradient boosting machine				
		Neural network				
Solana-Lavalle et al. [41]	64 HS	SVM, kNN, MLP, RF	8 to 20	0.95	0.93	0.96
	188 PD	baseline features, MFCC				
		WT features, TQWT features				
Sakar et al. [42]	64 HS	SVM, Naive Bayes	50	0.84	-	0.83
	188 PD	Logistic regression, kNN				
		Multilayer perceptron, RF				
Rana et al. [37]	48 HS	SVM	-	0.97	0.93	-
	147 PD	Naive Bayes				
		kNN, ANN				
Alrosan et al. [43]	8 HS	SVM	24	0.948	-	97.0
	23 PD	Logistic regression				
		kNN, RF				
Hoq et al. [46]	107 HS	MLP, kNN	-	0.935	-	0.951
	87 PD	RF, XGBoost				
		Sparse autoencoder + SVM				
Benba et al. [38]	20 HS	SVM	-	0.87	0.85	-
	20 PD	kNN				
Erdogdu et al. [44]	8 HS	VM, RF, kNN	22	0.964	-	0.96
	65 PD	Logistic Regression				
Parisi et al. [47]	40 HS	Gradient boosting, bagging	22	0.812	-	0.816
	40 PD	XGBoost				
		Extra tree classifier				
Costantini et al. [45]	266 HS	SVM, kNN	12 to 100	0.82	0.79	0.80
	160 PD	Naive Bayes				
Our proposal	44 HS	SVM, XGBoost	28	**0**.**98**	**0**.**99**	**0**.**98**
	28 PD	DT, RF, kNN				
	50 MS					

Our proposal involves working with two different diseases (i.e., PD and MS) and presents the best performance metrics (in bold)

While many studies investigate the application of machine learning techniques to vocal-based disease classification, particularly for Parkinson’s disease (PD), to the best of our knowledge, our proposal offers a novel contribution by examining a dataset comprising of both healthy subjects and individuals with two distinct pathologies (i.e., either PD or MS). This presents a more challenging scenario than the classic binary classification tasks commonly found in the literature. Contributions regarding multi-classification tasks have been proposed for voice disorders [73, 74] but, to the best of our knowledge, no contribution has so far addressed multi-classification in the context of neurodegenerative disease.

Our findings demonstrate the ability of machine learning techniques to effectively discriminate between healthy individuals and patients affected by one of the two pathologies, despite the heterogeneity of the feature space. This result underscores the robustness of our methodology and its potential use for real-world applications. Our approach can also make a valuable contribution to improving the multi-class classification of neurological disorders.

Our results align with previous studies highlighting the effectiveness of support vector machines (SVMs) for vocal-based disease classification. SVM emerges as a dominant classifier for vocal-based PD detection. For PD, cepstral-based features (e.g., MFCCs) prove to be highly effective in capturing disease-specific vocal alterations. In contrast, MS detection studies often emphasize the significance of formant-based features and fundamental frequency variations. This suggests that the pathophysiological mechanisms of these two diseases operate differently in the acoustic domain. However, our study makes a further contribution to the field by improving performance metrics, suggesting that our feature extraction and classification approach offers certain advantages over existing methods.

A key innovation of our work lies in the comprehensive analysis of both vowel and speech segments, allowing us to extract features from multiple domains. This approach enables us to achieve a more holistic representation of the vocal signal and enhances the accuracy of our classification models. To the best of our knowledge, few studies have explored this combined approach so far, particularly in the context of multi-class disease classification (as discussed in Sect. 2).

The proposed methodology presents limitations regarding the data acquisition process. We used two datasets: one available online and the other obtained by the clinical research unit. The acquisition process is heavily dependent on the capacity of acquiring vocal and speech signals from patients. Indeed, without the support of clinicians, it may be difficult to acquire datasets associated to pathologies in other settings. Furthermore, a data enrichment process is required to improve the quantity of homogeneous data in order to properly balance the different pathological classes. We claim that increasing open access to vocal datasets may promote the research on more reliable mechanisms for voice-related signal analysis as an early detection tool for general purpose neurological diseases identification.

In sum, our study provides valuable evidence on the feasibility of using machine learning techniques on vocal biomarkers to discriminate between healthy individuals and individuals with neurological disorders. Our methodology contributes novel insights to the field of vocal-based disease diagnosis by exploring the multi-class classification problem and deploying a multi-domain feature extraction approach. Future research will focus on (i) validating our findings on larger and more diverse datasets, (ii) investigating the potential of deep learning models to enhance classification, and (iii) exploring the integration of vocal biomarkers with other clinical data (e.g., genetic or imaging data) for a more comprehensive assessment of the progression of the disease.

## Conclusions

We proposed a novel voice-based methodology for disease classification, distinguishing between healthy individuals and patients with PD and MS. Our methodology implements machine learning techniques and a comprehensive feature extraction process to achieve accurate results in classifying two distinct neurological conditions. Key findings include the effectiveness of the XGBoost ML algorithm for vocal-based detection and the importance of cepstral-based features in capturing disease-specific vocal alterations.

We use an integrated approach which integrate both vowel and speech segments, providing a more holistic analysis of the vocal signal, leading to an improved classification for the two considered different diseases. Available tools use only formant-based features for the detection of a single disease. Moreover, an explainability module supporting ML results is also included to support physicians in the vocal biomarkers classification. Python codes and anonymized data set are available at (under request) https://github.com/PatriziaVizza/SpeechAndVocalSignalsAnalysis.

Future works include the possibility of validating our findings on larger and more diverse datasets, exploring the potential of deep learning models, and integrating vocal biomarkers with other clinical data for a more comprehensive assessment of disease progression.

## Data Availability

No datasets were generated or analysed during the current study.

## Footnotes

1 In Fourier analysis, the cepstrum is the result of computing the inverse Fourier transform (IFT) of the logarithm of the estimated signal spectrum.

## Code Availability

Code made available upon request.
